# Multisensory integration using dynamical Bayesian networks

**DOI:** 10.3389/fncom.2015.00058

**Published:** 2015-05-22

**Authors:** Taher Abbas Shangari, Mohsen Falahi, Fatemeh Bakouie, Shahriar Gharibzadeh

**Affiliations:** ^1^Amirkabir Robotic Center, Amirkabir University of TechnologyTehran, Iran; ^2^Institute for Cognitive and Brain Sciences, Shahid Beheshti UniversityTehran, Iran

**Keywords:** multisensory integration, Dynamic Bayesian Networks, modeling, sensory processing disorder, Bayesian Models

Multisensory Integration (MSI) is the study of how information coming from different sensory modalities, such as vision, audition and etc. are being integrated by the nervous system (Stein et al., 2009) as a complex system. MSI is one of the most important aspects of neuroscience which has a great influence on our decision making system. It plays a key role in our understanding of surrounding environment which makes a coherent representation of the world for us (Lewkowicz and Ghazanfar, 2009). Since signals in our sensory systems are corrupted by variability or noise, the nervous system combines different kinds of sensory information like sound, touch etc. to achieve a meaningful and continuous stream of percepts (Kording and Wolpert, 2006; Lewkowicz and Ghazanfar, 2009). Recently, researchers have shown an increased interest in MSI modeling, to discover the causes of related disorders such as under-sensitivity or hyposensitivity (Knill and Pouget, 2004). Moreover individuals with Autism Spectrum Disorder (ASD) have an impaired ability to integrate multisensory information to make a unified percept (Stevenson et al., 2014).

Different researches have modeled MSI in a variety of ways. Computational methods, such as Kalman Filter (KF) and Bayesian Networks (BN) are used widely to model probabilistic functions of the nervous system including MSI (Van Der Kooij et al., 1999; Kording and Wolpert, 2004). In KF-based models there is a basic assumption on accuracy of the sensory input data. This assumption says that the error's Probability Density Function (PDF) of each sensor is Gaussian. According to KF, it is provable that data fusion of two different kinds of data for one variable measurement leads to more accurate results (Kalman, 1960). A serious weakness with this method, however, is its basic assumption. Assuming a Gaussian form of the PDF of the sensory systems' error is in contradiction with the brain's internal models and prior knowledge about human sensory system and environmental models which are not necessarily Gaussian-like. Additionally, as different formats are used by each sensory modality to encode the same properties of the environment or body, MSI cannot be as simple as an averaging between sensory inputs (Deneve and Pouget, 2004). Hence, it is clear that KF-based models are not valid for many MSI studies and therefore researchers tried to modify this method (Van der Zijpp and Hamerslag, 1994; Julier and Jeffrey, 2004).

Since BNs have not any assumption on accuracy of the input data, they have attracted much attention recently. A BN is a graphical model that represents probabilistic relationships among variables of interest. By using graphical models in conjunction with statistical techniques, several advantages for data analysis will be obtained: Firstly, because a BN represents conditional dependencies among all variables, it is able to handle situations where some data entries are missing. Secondly, the model can be used to learn causal relationships, so it can be used to understand a problem domain and to predict the consequences of intervention. Thirdly, because BNs have both causal and probabilistic semantics, they represent combining prior knowledge and data ideally (Heckerman, 1998; Wasserman, 2011).

Generally, there are three main inference tasks for BNs: inferring unobserved variables, parameter learning, and structure learning. They are used widely for modeling knowledge in computational biology, bioinformatics, etc. For example, a BN could represent the probabilistic relationships between diseases and symptoms. Given symptoms, the network can be used to compute the probabilities of the presence of various diseases.

As it mentioned before, the brain needs using different resources of information altogether to be able to make a sound decision about a situation. In such cases BNs can be used to model brain's function in many studies (Seilheimer et al., 2014). It is worth mentioning that in BNs, relationship between different nodes is not as simple as an averaging and we can model more complex probabilistic problems by using BNs (Bishop and Nasser, 2006).

However, it is obvious that the reliability of sensory modalities varies widely according to the context and in a BN the effect of one node on the other one can vary from one task or situation to another one. But it is clear that when we assume a node as a parent node for another one, this relation could not be changed and new experiences would not cause new links between separated nodes. The main weakness of the BN based models is the failure to address the way it uses to reconstruct the network, based on new observed experiences. Most studies in MSI modeling have only focused on one task in which the effective sensory resources are known before, therefore, the structure of the network is known too, and we only need to train the network. By contrast, when we want to model MSI, we should not restrain it only in some certain tasks but the model should instead be generalizable to other tasks. It means that the model should be more dynamic and task independent. In addition, it is clear that time has a great influence in our decision making and reasoning and unfortunately, BN fails to code the time directly (Mihajlovic and Petkovic, 2001).

We suggest that, MSI models will be more generalized if we use Dynamic Bayesian Networks (DBN) which describes a system that dynamically changes over time. In a BN that models the interactions between sensory modalities, the nodes are associated with activated sensory modalities and the edges represent the interactions among sensory modalities. Sensory modalities of a neural system including n sensory modalities are indexed in a set *I* = {*i*: *i* = 1, 2, … *n*}. Consider activation of a sensory modality measured by fMRI time-series or EEG over the sensory modality. Let *x_i_* be the activation measuring the response of sensory modality *i*.

BNs describe the PDF over the activation of sensory modalities, where the graphical structure provides an easy way to specify conditional interdependencies for a compact parameterization of the distribution. A BN defined by a structure *S* is a directed acyclic graph (DAG) and a joint distribution over the set of time-series *x* = {*x_i_*: *i* ∈ *I*}. The set of activations of the parents of sensory modality *i* is denoted by *a_i_*, and a DAG offers a simple and unique way to decompose the likelihood of activation in terms of conditional probabilities: where θ = {θ_*i*_: *i* ∈ *I*} represents the parameters of the conditional probabilities (Rajapakse and Zhou, 2007).

DBNs extend BNs to incorporate temporal characteristics of the time-series *x*. *x*(*t*) = {*x_i_*(*t*): *i* ∈ *I*} represents the activations of *n* sensory modalities at time *t*, where the instances *t* = 1, 2, … *T* correspond to the times when sensory modality measures are taken and *T* denotes the total number of measures. In order to model the temporal dynamics of brain processes, we need to model a probability distribution over the set of random variables $\cup_{t=1}^{T}x(t)$ which is complex and practically hard.

To avoid an explosion of the model complexity, one can assume that the temporal changes of activations of brain regions are stationary and first-order Markovian. This assumption provides a tractable causal model that explicitly takes into account the temporal dependencies of brain processes. When facing more complex temporal processes and connectivity patterns, higher-order and non-stationary Markov models can be used to overcome the complexity.

The connectivity structure between two consecutive data sampling is represented by the transition network, which renders the joint distribution of all possible trajectories of temporal processes. The structure of the DBN is obtained by unrolling the transition network over consecutive scans for all *t* = 1, 2, …, *T* (Rajapakse and Zhou, 2007).

In an overview, we here suggest that DBN may be a more useful method to model MSI in comparison to prior methods because of three reasons. Firstly, as DBN changes dynamically, initial structure of the network does not lead to an unreliable result and we can use the network in various kinds of studies (because this method is task-independent). Secondly, in cases which we are not sure about the relation and interaction between different sensory modalities, DBN output can help us to achieve a more accurate understanding about MSI processes. Moreover, there exist cyclic functional networks in the brain, such as cortico-subcortical loops which BNs are not capable to model. Unlike BN, DBN has the capability of modeling recurrent networks while still satisfying the acyclic constraint of the transition network (Rajapakse and Zhou, 2007). This is an important advantage of modeling neural system with DBN as these key features of DBN help us to obtain a proper viewpoint about MSI in different tasks and it makes the study of related disorders easier and closer to reality.

## Conflict of interest statement

The authors declare that the research was conducted in the absence of any commercial or financial relationships that could be construed as a potential conflict of interest.
